# The evolutionary landscape and serotypic dynamics of avian infectious bronchitis virus from spike protein

**DOI:** 10.1002/imo2.70077

**Published:** 2026-01-19

**Authors:** Hao Zhang, Zongxi Han, Chuangchao Zou, Yihui Huang, Shiping Sun, Ouyang Peng, Usama Ashraf, Qiuping Xu, Yao Ge, Qixiang Kang, Xiuli Ma, Xinheng Zhang, Ye Zhao, Xin Yang, Hongning Wang, Guozhong Zhang, Qingmei Xie, Mang Shi, Yongsen Ruan, Min Liao, Shengwang Liu, Yongchang Cao

**Affiliations:** ^1^ State Key Laboratory of Biocontrol, School of Life Sciences Sun Yat‐sen University Guangzhou China; ^2^ State Key Laboratory of Animal Disease Control and Prevention, Harbin Veterinary Research Institute Chinese Academy of Agricultural Sciences Harbin China; ^3^ Key Laboratory of Animal Virology of Ministry of Agriculture Zhejiang University Hangzhou China; ^4^ Programme in Emerging Infectious Diseases Duke‐NUS Medical School Singapore Singapore; ^5^ Guangdong Provincial Key Laboratory of Malignant Tumor Epigenetics and Gene Regulation, Sun Yat‐sen Memorial Hospital Sun Yat‐sen University Guangzhou China; ^6^ Shandong Academy of Agricultural Sciences Jinan China; ^7^ College of Animal Science South China Agricultural University Guangzhou China; ^8^ College of Veterinary Medicine China Agricultural University Beijing China; ^9^ College of Life Sciences Sichuan University Chengdu China; ^10^ Key Laboratory of Intelligent Tracking and Forecasting for Infectious Diseases, State Key Laboratory for Biocontrol, School of Medicine Shenzhen Campus of Sun Yat‐sen University, Sun Yat‐sen University Shenzhen China

**Keywords:** coronavirus, evolution, N‐linked glycosylation, transmission, serotype prediction

## Abstract

Avian infectious bronchitis virus (IBV), a gammacoronavirus with substantial agricultural impact, offers a tractable model for dissecting coronavirus evolution. Here, we integrated 20 years of epidemiological surveillance with whole‐genome sequence analysis of 624 IBV strains, including 136 newly isolated field samples, to investigate the evolutionary and structural dynamics of N‐linked glycosylation at the spike protein. We identified three dominant glycosylation haplotypes defined by residues 51 and 77 of spike protein, which correlate with receptor‐binding interfaces, clinical phenotypes, and spatiotemporal transmission patterns. Molecular modeling and docking analyses provided insights into potential mechanistic links between glycan positioning and Neu5Acα2‐3Galβ1‐3GlcNAc receptor engagement. Complementing these findings, we developed a proof‐of‐concept machine learning model that shows potential for predicting clinical serotypes directly from the spike protein sequence, achieving high accuracy on a preliminary independent validation set. These findings support the use of glycosylation motifs as structural‐genomic markers and highlight the potential of sequence‐based serotype prediction. Our work establishes a scalable genomic‐structural framework that leverages glycosylation motifs and sequence features as evolutionary markers, providing a powerful approach for forecasting coronavirus adaptation and informing vaccine design and outbreak preparedness.

## Highlights

- Epidemiological surveillance and evolutionary analysis reveal the complex spatiotemporal transmission dynamics of avian infectious bronchitis virus (IBV) across the whole world.
- Key spike protein glycosylation haplotypes are associated with receptor‐binding specificity and are linked to distinct clinical outcomes.
- Glycosylation motifs serve as structural‐genomic markers, offering a scalable framework for predicting coronavirus evolution and spread.
- Spike protein‐based machine learning method enables reliable prediction of IBV serotypes.

## Graphical abstract

This study integrates two decades of surveillance with genomic and structural analyses to decipher how spike protein glycosylation haplotypes drive avian coronavirus evolution. We uncover how specific glycosylation patterns associate with receptor‐binding affinity, shape global transmission dynamics, and correlate with clinical outcomes. Furthermore, we develop a machine learning framework for highly reliable serotype prediction directly from spike protein sequences. This multidisciplinary work establishes a scalable “viral genomic‐structural‐phenotypic” framework to forecast coronavirus evolution and guide vaccine design.

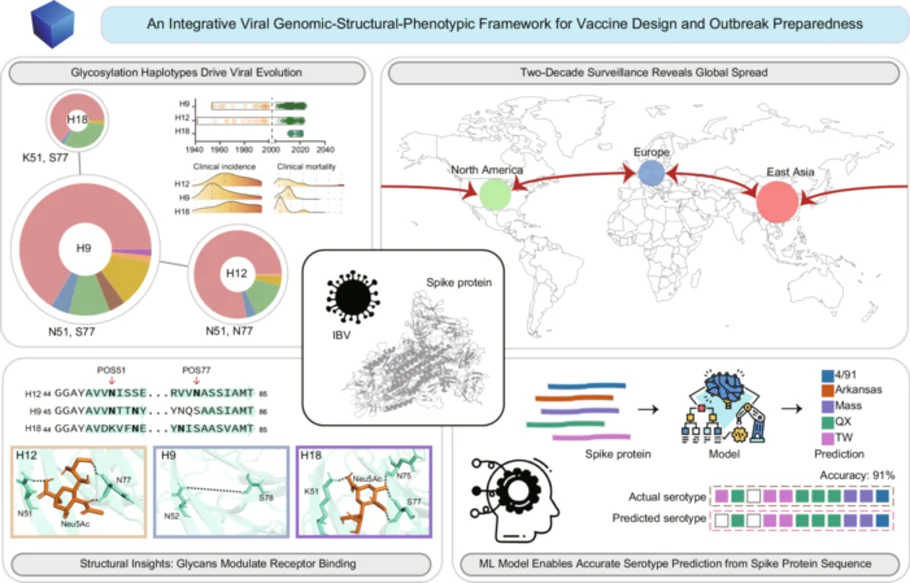

## 1 INTRODUCTION

The COVID‐19 pandemic has underscored the critical need to understand how coronaviruses evolve, spread, and adapt in both animal and human populations, which is crucial for maintaining public health. So far, global efforts have mainly focused on human‐pathogenic coronaviruses such as SARS‐CoV‐2; however, other zoonotic coronaviruses circulating in livestock and wildlife represent a persistent threat to the human population due to an undercharacterized reservoir of their genetic diversity [1, 2]. Among these, avian infectious bronchitis virus (IBV), the first discovered coronavirus and a significant pathogen in poultry, offers a valuable comparative model for studying long‐term coronavirus evolution, antigenic diversification, and epidemic dynamics under natural and vaccine‐driven selection pressures [3, 4]. IBV has circulated globally since its discovery nearly a century ago, causing significant morbidity and mortality in chickens and posing a continuing threat to food security [5]. Like other coronaviruses, IBV exhibits high mutation and recombination rates, and its rapid antigenic drift has repeatedly undermined vaccination efforts [6]. Besides, previous studies have highlighted the pivotal role of the IBV spike protein (S protein) in genotyping and serotyping of IBV, with subtle sequence variations potentially influencing serotype shifts [7]. Although IBV has circulated under intensive surveillance in high‐density poultry systems for decades, its potential as a model to explore broader questions of coronavirus adaptation, dissemination, and immune escape remains underexploited. A critical and persistent challenge in this endeavor is the accurate prediction of serotypes, which are crucial for vaccine matching but are difficult to determine rapidly due to the complex and labor‐intensive nature of serological assays.

The coronavirus spike (S) protein, a principal target of neutralizing antibodies, is a heavily glycosylated trimeric glycoprotein that plays a critical role in mediating viral entry and immune evasion [8]. N‐linked glycans on the S protein form a dynamic shield that masks key antigenic epitopes, thereby reducing recognition by host antibodies [9, 10].  Structural studies have identified >60 glycosylation sites on the S trimer of SARS‐CoV, MERS‐CoV, and SARS‐CoV‐2, which highlight conserved and extensive glycan modification of the coronavirus spike protein [11]. These glycan shields are strongly associated with viral immune escape and modulate the host neutralizing antibody responses [9]. Despite their established importance in other coronaviruses, the evolutionary dynamics of glycosylation in IBV and their potential roles in antigenic variation or virulence remain poorly understood. To date, systematic analyses linking spike glycosylation site evolution to IBV immune escape or pathogenicity are lacking. Beyond glycosylation, the spike protein itself is the primary determinant of antigenic specificity and serotype in IBV [7]. Besides, glycan positioning can alter epitope exposure and receptor‐binding configuration [9], and thus, its evolution may underlie serotype diversification. However, predicting serotype from sequence data has been challenging, hindering rapid surveillance and vaccine selection.

To address this gap, we conducted a longitudinal genomic surveillance study over two decades, sequencing 136 newly isolated IBV genomes from clinical poultry cases and integrating them with publicly available IBV genomic datasets. Phylogenetic reconstruction revealed a complex evolutionary landscape and distinct patterns of mutation and recombination. We performed a haplotype‐level analysis of N‐linked glycosylation sites within the IBV spike protein, accompanied by structural modeling of sialic acid‐binding interactions. It allowed us to construct the first glycosylation haplotype network for IBV and uncover a strong association between glycan‐site evolution, receptor‐binding conformation, and viral phenotype. Furthermore, to tackle the challenge of serotype prediction, we developed a novel machine learning model that achieves high accuracy in predicting clinical serotypes directly from spike protein sequences. Together, these findings significantly advance our understanding of the clinical epidemiology and evolution of coronaviruses, providing a foundation for the genotyping, characterization, and effective control of IBV.

## 2 RESULTS

### Epidemic of avian viruses in China during the last two decades (2003–2023)

A nationwide surveillance program, spanning two decades (2003–2023), systematically monitored the prevalence of six major avian viral pathogens [IBV, avian influenza virus (AIV), avian reovirus (ARV), infectious bursal disease virus (IBDV), Newcastle disease virus (NDV), and infectious laryngotracheitis virus (ILTV)] across poultry farms affected by avian diseases in China (Figure 1A–F, Table S1). By conducting >1 million diagnostic assays, a clear dominance of IBV was observed, which accounted for 462,290 diagnostic assays and represented the highest incidence among all surveyed viruses. Temporal dynamics in IBV prevalence and mortality were notable: peak positivity and mortality rates reached ~50% and 15%, respectively, in 2009, but declined to below 30% and 11%, respectively, after 2020. Among all viruses, IBV exhibited the highest mean positivity (38.5%) and mortality (11.0%) rates, exceeding ILTV, which was the second most prevalent pathogen with positivity and mortality rates of 31.1% and 6.0%, respectively. In contrast, the remaining viruses exhibited substantially lower mean positivity (11.7–28.9%) and mortality (1.7–3.6%) rates. These findings indicate IBV as a dominant epidemiological agent within this cohort, warranting a deeper molecular investigation. To this end, we performed whole‐genome sequencing of 136 IBV isolates collected over multiple years and across diverse geographic regions in China (Figure S1). All sequences have been deposited in the NCBI GenBank database (Table S2) and are publicly available.

**FIGURE 1:**
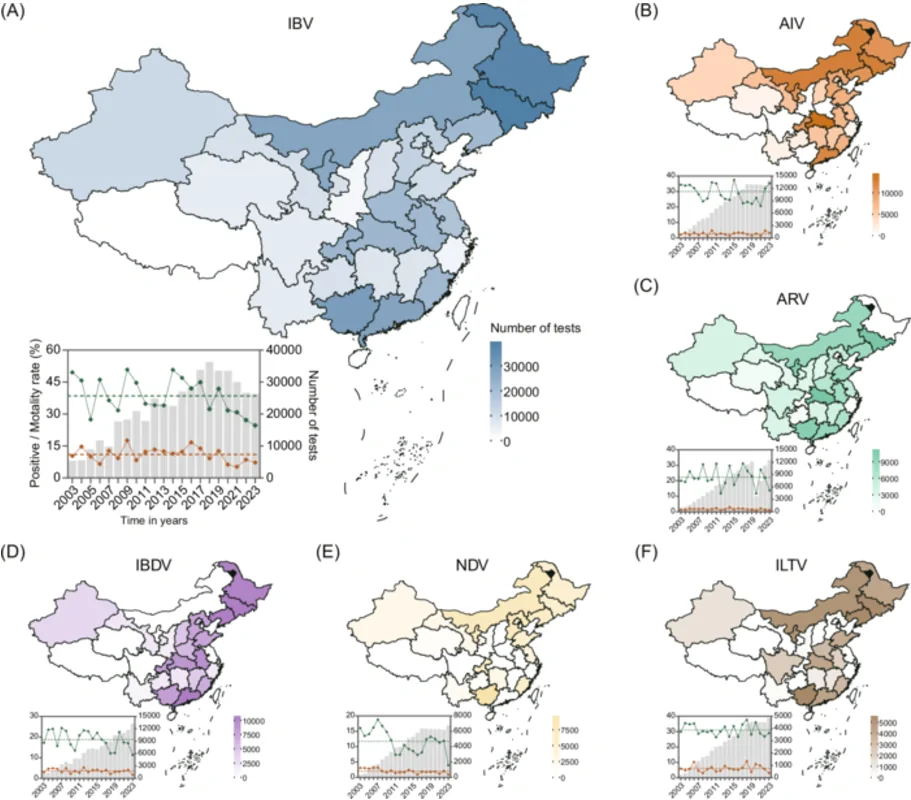
Spatiotemporal patterns of major avian viral pathogens in China, 2003–2023. Geographic and temporal distributions of six economically significant avian viruses: infectious bronchitis virus (IBV) (A), avian influenza virus (AIV) (B), avian reovirus (ARV) (C), infectious bursal disease virus (IBDV) (D), Newcastle disease virus (NDV) (E), and infectious laryngotracheitis virus (ILTV) (F). Choropleth maps indicate the cumulative number of diagnostic tests performed per province. Line graphs below each map depict longitudinal trends in positivity rate (green line, left *y*‐axis), mortality rate (orange line, left *y*‐axis), and total number of annual diagnostic tests (gray bars, right *y*‐axis). The *x*‐axis denotes the calendar year of sample collection, spanning 2003 to 2023.

### Complex phylogeny, homology, and evolutionary dynamics of IBV strains

To investigate the phylodynamic evolution of IBV, we expanded our genomic dataset beyond the 136 newly sequenced Chinese isolates by integrating 488 publicly available whole‐genome sequences from the NCBI database, resulting in a comprehensive global cohort of 624 strains (Table S2). These sequences span 32 countries across five continents, with Chinese‐origin strains constituting the majority (69%; *n* = 433), followed by contributions from the United States and Mexico (Figure 2A). For comparative analyses, the dataset was partitioned into seven geographically defined subsets: China, Europe, North America, South America, other Asian countries, Africa, and Australia.

**FIGURE 2:**
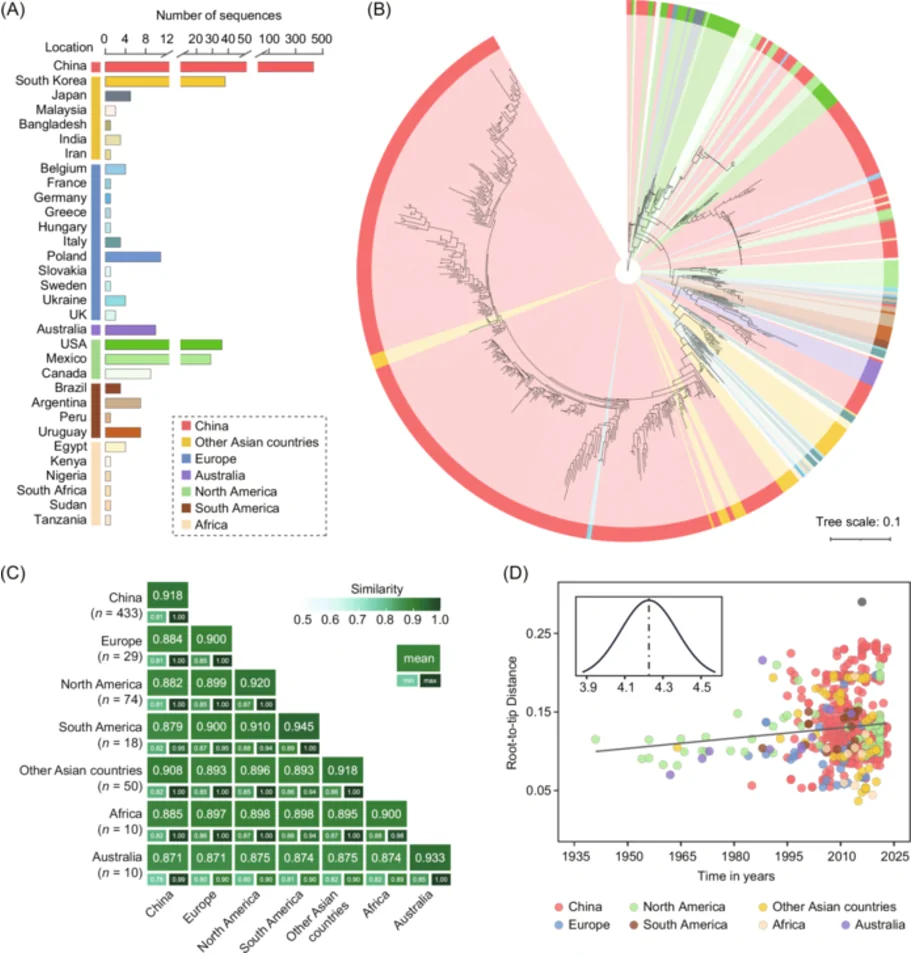
Global genetic diversity and evolutionary dynamics of IBV. (A) Geographic origin of 624 complete IBV genome sequences, categorized by country and seven defined geographic regions: China, Europe, North America, South America, other Asian countries, Africa, and Australia. (B) Maximum likelihood phylogenetic tree inferred from all 624 complete genomes, illustrating the global diversity and structure of IBV lineages. Leaf colors correspond to the different countries, following the same color scheme as in panel (A). (C) Pairwise nucleotide similarity matrix of complete IBV genomes across seven geographic regions. The corresponding mean, minimum, and maximum values were calculated from all pairwise sequence comparisons between the two respective geographic regions. (D) Root‐to‐tip genetic distances plotted against sampling dates, along with the estimate of substitution rates, using 624 complete IBV genome sequences. The strains (each dot represents a strain) in the outer plot were included in the linear fitting of the evolutionary rate estimation according to TreeTime [12]. The inner plot shows the posterior probability densities of the mean estimated evolutionary rate, and the *x*‐axis represents the evolutionary rate (×10^−4^ substitutions per site per year). Colors correspond to the seven geographic regions, following the same color scheme as in panel (A).

Maximum‐likelihood phylogenetic reconstruction revealed partial geographic structuring across the global collection (Figure 2B). Early‐diverging lineages were predominantly composed of Chinese isolates, while distinct clades were formed by sequences from Australia and South America. Notably, basal phylogenetic nodes showed admixture between Chinese and North American strains, suggesting historical intercontinental exchange during the early diversification of the virus.

Pairwise nucleotide identity comparisons across regional subsets revealed whole‐genome similarities ranging from 0.871 to 0.910 (Figure 2C). Chinese sequences exhibited the highest genetic affinity with those from other Asian countries (mean identity: 0.908), whereas Australian isolates showed the lowest inter‐regional similarity (mean identity: 0.871–0.875). Divergence patterns observed across the whole genome were broadly mirrored in ORF1a/b and structural gene regions, including the envelope (E), membrane (M), and nucleocapsid (N) genes. Notably, the S gene displayed the highest degree of sequence divergence among all genomic regions, underscoring its central role in viral diversification (Figure S2).

Molecular clock analysis estimated a global substitution rate of 4.23 × 10^−4^ substitutions per site per year (Figure 2D). However, considerable regional variation was evident: strains from Africa (1.33 × 10^−3^), other Asian countries (1.12 × 10^−3^), and China (1.05 × 10^−3^) exhibited higher evolutionary rates, whereas strains from North America (3.11 × 10^−4^), Australia (3.10 × 10^−4^), Europe (2.30 × 10^−4^), and South America (1.22 × 10^−4^) evolved at slower rates. (Figure S3 and Table S3). Recombination analysis further revealed recurrent breakpoint hotspots concentrated in the S gene and ORF1a region across all geographic subsets (Figure S4 and Table S4). Together, these findings highlight the intricate spatiotemporal dynamics and region‐specific adaptive strategies that shape the global evolutionary landscape of IBV.

### Haplotype dynamics of N‐glycosylated sites within the spike (S) protein

In light of the extensive evolutionary variability observed across full‐length IBV genomes and structural genes, we focused on N‐linked glycosylation within the S protein, which is a key determinant of viral entry and immune evasion [9, 11, 13, 14]. Analysis of 10 predicted N‐glycosylation sites in the N‐terminal domain (NTD) across 623 strains showed pronounced position‐specific heterogeneity. Asparagine (Asn) was the predominant residue at nearly all glycosylation sites (Figure 3A, Table S5). Based on the M41 reference strain (GenBank accession Number DQ834384), position 51 exhibited the highest amino acid diversity, with Asn present in 86.0% of strains and seven alternative residues detected. In contrast, position 77 demonstrated a strong preference for serine (Ser; 71.7%). All remaining positions exhibited >91.8% conservation of Asn.

**FIGURE 3:**
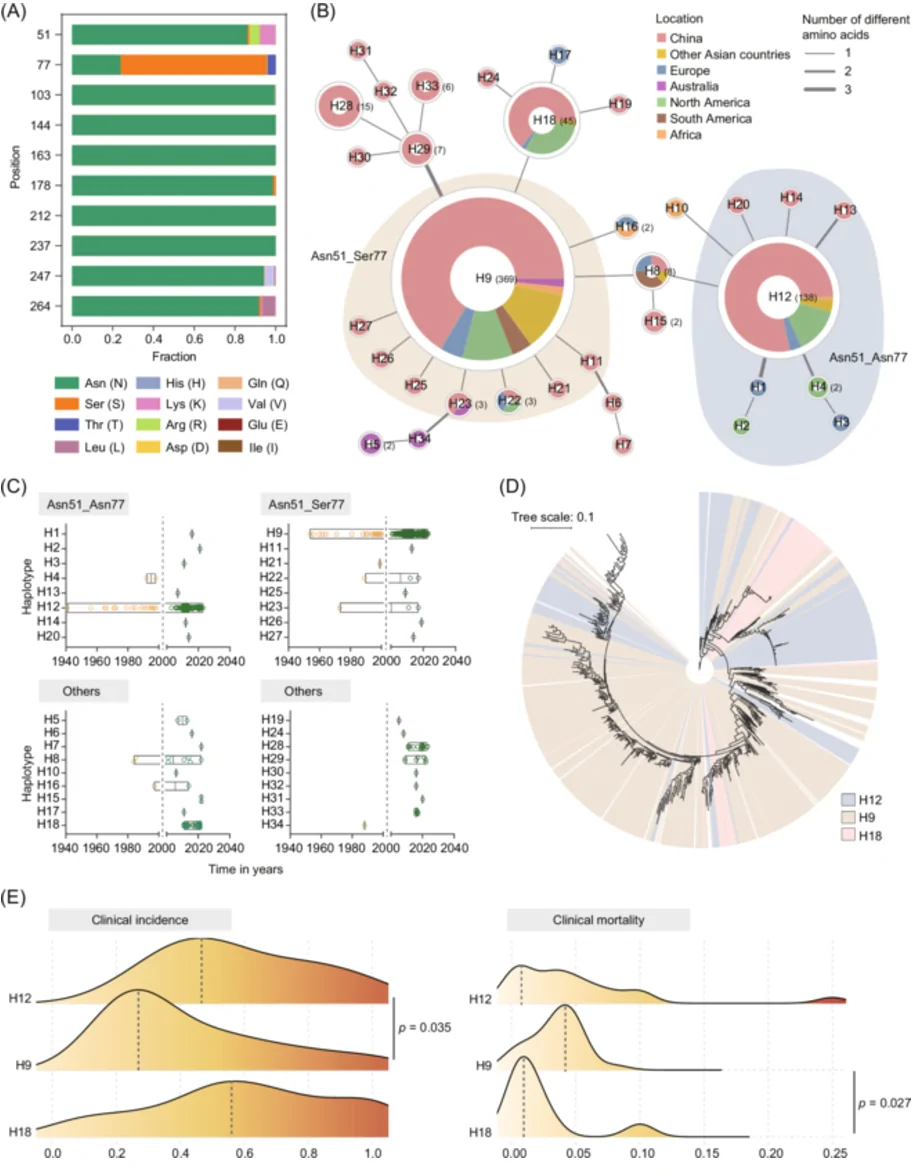
Evolutionary diversification of N‐linked glycosylation haplotypes in the N‐terminal domain (NTD) of IBV S protein. (A) Amino acid distribution across 10 predicted N‐linked glycosylation sites in the NTD of IBV spike protein. All annotated positions are relative to the M41 reference strain (GenBank accession Number DQ834384). (B) Median‐joining haplotype network constructed from 623 IBV S sequences based on the 10 N‐linked glycosylation sites. Each circle represents a unique haplotype; circle size indicates the number of sequences (number shown in parentheses if >1), and colors denote geographic origins. Edge thickness corresponds to the number of amino acid substitutions between haplotypes. (C) Temporal distribution of sequences assigned to each glycosylation haplotype. Isolates collected before 2000 were depicted in orange, and isolates collected after 2000 were depicted in green. (D) Maximum likelihood phylogenetic tree inferred from all 624 complete genomes. Strains of the three major haplotypes (H12, H9, and H18) were depicted in different colors. (E) Comparison of clinical positivity and mortality rates among the three major haplotypes (H12, H9, and H18). Statistical comparisons were conducted using the Kolmogorov–Smirnov test; *p*‐values < 0.05 are annotated. Exact values of clinical positivity and mortality rates are listed in Table S2.

Haplotype network reconstruction based on these NTD glycosylation patterns identified 34 distinct profiles (Figure 3B). Three dominant haplotypes, H9 (*n* = 369), H12 (*n* = 138), and H18 (*n* = 45), accounted for 86% of all strains. Notably, these three haplotypes were distinguished solely by residues at positions 51 and 77, while the remaining eight sites retained conserved Asn residues. Haplotypes were categorized into two major types: Asn51_Asn77 and Asn51_Ser77. Isolates collected before 2000 belonged to either H9 or H12, with H12 containing the prototypic M41 strain, representing the most ancestral form (Figure 3C). The topology of the haplotype network showed a strong concordance with temporal phylogeny, with root‐proximal nodes corresponding to early sampling dates (Figure S5). Furthermore, we mapped the three major haplotypes (H12, H9, and H18) onto the whole‐genome phylogeny (Figure 3D). Despite their high overall frequency, both H9 and H12 were observed to have emerged independently within multiple and distinct lineages.

Clinical association analyses suggested phenotypic divergence among haplotypes: strains belonging to H9 exhibited significantly lower positivity rates compared to H12 (Kolmogorov–Smirnov test, *p* = 0.035), yet displayed higher mortality rates than H18 strains (*p* = 0.027) (Figure 3E). Although other comparisons did not reach statistical significance, these findings suggest that variation in glycosylation site composition, particularly at residues 51 and 77, may be associated with differences in pathogenicity.

A parallel haplotype analysis targeting 14 predicted N‐glycosylation sites within the S2 subunit identified position 542 and 686, proximal to the furin cleavage sites, as sites of notable conservation, wherein Asn and Ser predominate at frequencies of 83.8% and 89.2%, respectively (Figure S6A). These residues were consistently conserved across ancestral haplotypes, including H10, H12, and H13 (Table S5). Variants at position 542 (e.g., Ser542 and Thr542) formed distinct haplotype clusters, as did variants with Asn686 at position 686 (Figure S6B). However, the comparison of clinical positivity and mortality rates among haplotypes carrying Asn542_Asn686, Asn542_Ser686, and Ser542_Ser686 combinations revealed no significant differences (Kolmogorov–Smirnov test, all *p* > 0.05) (Figure S6C), suggesting that glycosylation variants near the furin cleavage site may not exert strong phenotypic effects.

### The N‐glycosylation substitutions alter the docking of N‐acetylneuraminic acid

To elucidate the phenotypic consequences of N‐glycosylation at positions 51 and 77 within the NTD of the S protein, we selected three representative IBV strains corresponding to distinct glycosylation haplotypes: M41 (H12; GenBank accession Number DQ834384), ck/CH/LSD08‐7 (H9; KX302860), and ck/CH/LSD/110857 (H18; KP118885). Structural predictions using AlphaFold3 and molecular docking analyses targeting the Neu5Acα2‐3Galβ1‐3GlcNAc receptor (hereafter referred to as “the sialoside receptor”) were employed to evaluate receptor‐binding potential. Sequence analysis of N‐glycosylation motifs (Asn‐X‐Ser/Thr) within the NTD showed haplotype‐specific conservation patterns: M41 retained canonical glycosylation motifs at both positions 51 and 77; ck/CH/LSD08‐7 preserved the motif only at position 51 and presented non‐conservative substitutions at position 77; whereas ck/CH/LSD/110857 exhibited structurally analogous motifs at positions 54 and 75, instead of the canonical 51 and 77 sites (Figure 4A, Table S6).

**FIGURE 4:**
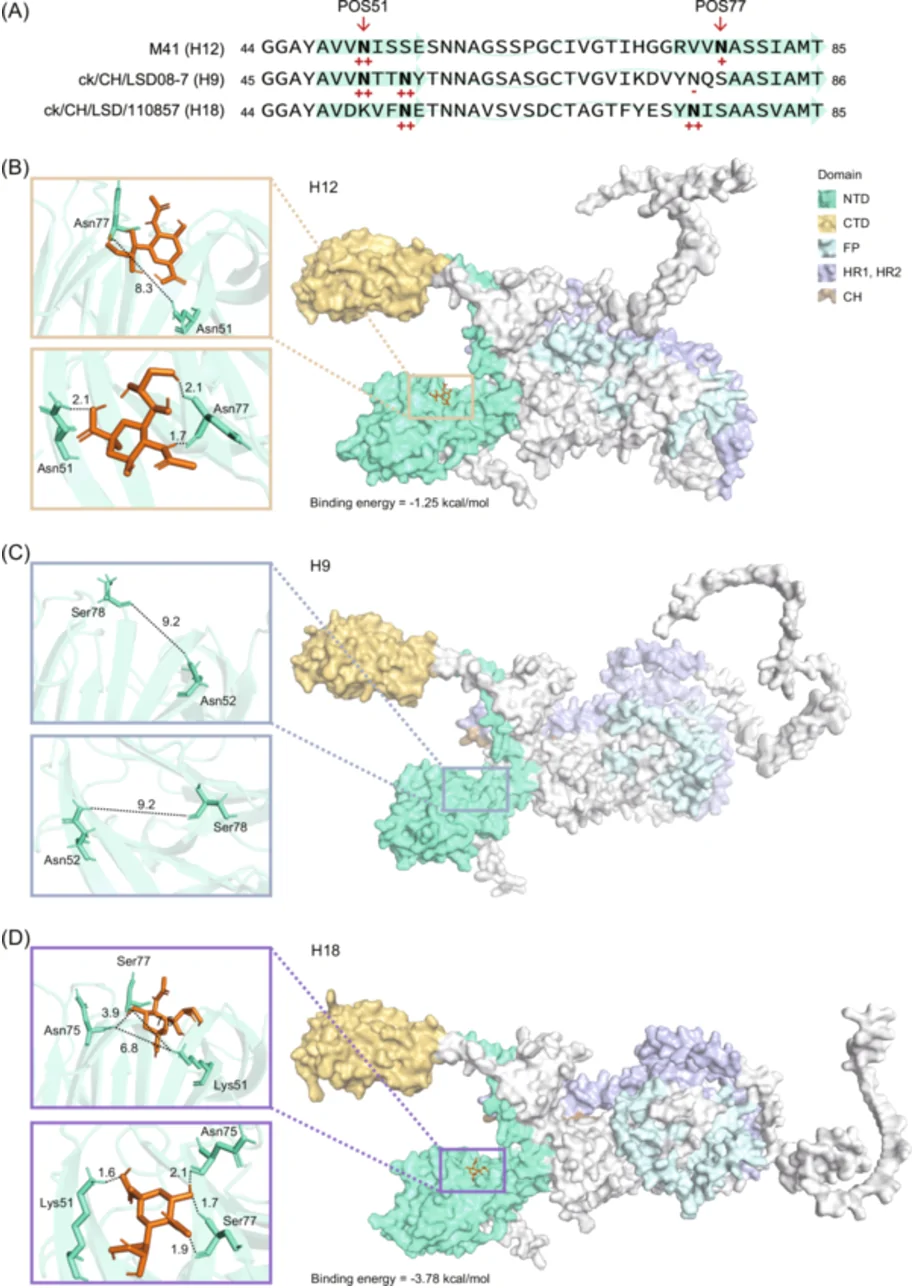
Structural insights into N‐glycosylation‐mediated modulation of receptor binding in IBV S NTD haplotypes. (A) Alignment of amino acid sequences near positions (POS) 51 and 77 of the S NTD for three representative haplotypes: M41 (H12; GenBank accession Number DQ834384), ck/CH/LSD08‐7 (H9; KX302860), and ck/CH/LSD/110857 (H18; KP118885). Predicted N‐linked glycosylation sites are highlighted in boldface (Asn residues), and confidence of glycosylation is indicated below each site (“+,” predicted glycosylated; “–,” predicted non‐glycosylated. A greater number of symbols denotes a higher prediction confidence), as determined by NetNGlyc 1.0 (https://services.healthtech.dtu.dk/services/NetNGlyc-1.0/). (B‐D) Alphafold3‐based predicted structural models of the S protein for strains of (B) M41 (H12), (C) ck/CH/LSD08‐7 (H9), and (D) ck/CH/LSD/110857 (H18), each showing molecular docking with the Neu5Acα2‐3Galβ1‐3GlcNAc receptor. Predicted docking poses are displayed, with binding affinities and inter‐molecular distances (black dashed line) annotated. CTD, C‐terminal domain; CH, central helix; FP, fusion peptide; HR1 and HR2, heptad repeats 1 and 2; NTD, N‐terminal domain.

AlphaFold‐based structural modeling demonstrated that glycosylation sites at these positions localize to solvent‐exposed β‐sheets across all three strains (Figure 4B–D). Docking analyses predicted differential Neu5Ac‐binding capabilities: M41 formed a putative binding pocket involving Asn51 and Asn77 (binding energy: –1.25 kcal/mol; three hydrogen bonds), whereas ck/CH/LSD/110857 suggested a potential binding affinity through a composite interface comprising Lys51, Asn75, and Ser77 (binding energy: –3.78 kcal/mol; four hydrogen bonds). By contrast, ck/CH/LSD08‐7 was predicted to lack the sialoside receptor‐binding potential at positions 51 and 77 due to disruption of the glycan‐interacting topology. These findings suggest that variation in glycosylation motif positioning can substantially alter receptor engagement.

We further investigated the structural consequences of N‐glycosylation near the two furin cleavage sites in the S2 subunit, specifically at positions 542 and 686. Notably, these residues lie within predicted epitope regions. Modeling indicated that glycan occupancy at these sites may modulate local structural stability at the cleavage sites, potentially influencing epitope architecture and immune recognition (Figure S7).

### Spatiotemporal reconstruction of IBV dissemination across the world

By integrating the haplotype network in NTD regions with discrete phylogeographic inference conducted via BEAST, we reconstructed the historical dissemination dynamics of IBV. Our analyses revealed that from the mid‐20th century through the early 2000s, North America, Europe, and East Asia, particularly China, served as persistent epicenters of transmission, forming a stable triangular axis that largely governed global viral dispersal (Figure 5A). Beyond this primary triad, additional transmission routes were observed, notably from North America to both Australia and South America, and from Europe to Africa. During the 2008–2011 period, IBV spread remained relatively constrained and stable (Figure 5B). In contrast, the years 2011–2017 marked a phase of rapid geographic expansion, characterized by intensified connectivity within the core triangle and markedly increased exchanges between South America, Africa, and other regions (Figure 5C). Since 2018, viral movement has predominantly refocused along the North America–Europe–East Asia corridor, which underscores the robustness of this transmission network despite ongoing diversification (Figure 5D).

**FIGURE 5:**
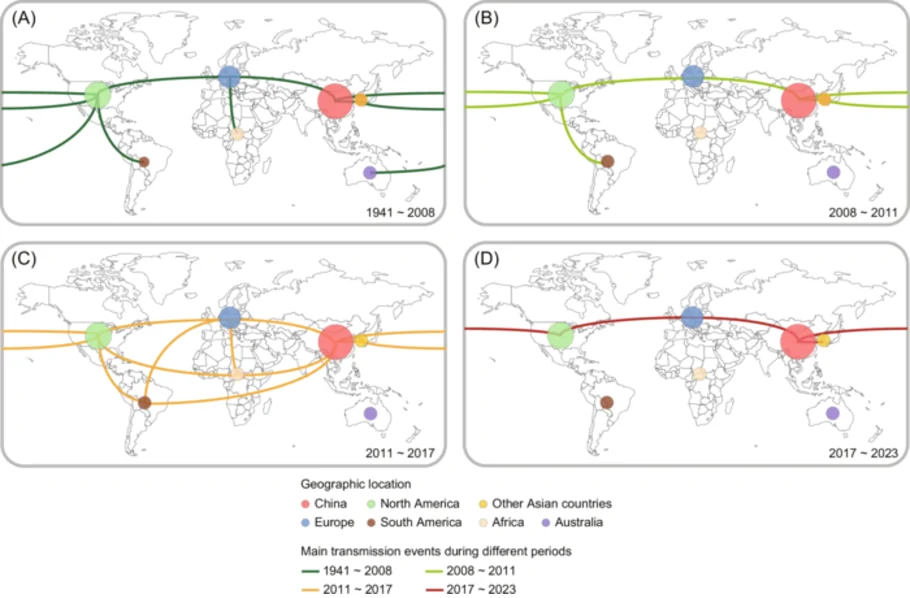
Spatiotemporal dissemination of infectious bronchitis virus (IBV) Strains globally. The Bayesian stochastic search variable selection approach was used to elucidate the spatiotemporal dynamics of IBV dissemination. The intensity of inferred transmission events, supported by a Bayes Factor exceeding three, is visualized. Color‐coding differentiates transmission events occurring across four distinct temporal periods from 1941 to 2008 (A), 2008 to 2011 (B), 2011 to 2017 (C), and 2017 to 2023 (D). The variation in circle size is set for visual distinction and carries no quantitative meaning regarding the geographic data.

Given that 433 of the 624 complete IBV genome sequences in our dataset originated from China (Figure 2A), we further reconstructed the domestic dissemination patterns of IBV within China (Figure S8). A time‐structured analysis showed that IBV in China primarily emerged in Jiangsu province in the 1980s and subsequently spread to other coastal and inland provinces. After its introduction to Shandong province in 1997, Shandong became the new epicenter of IBV dissemination, giving rise to numerous transmission chains from Shandong to other provinces over the following decade. IBV experienced rapid national dissemination between 2007 and 2017, and formed a more complex transmission network. Since 2017, most IBV outbreaks have been concentrated in the central provinces of China.

### Machine learning (ML)‐based IBV serotype model construction and validation

The serotyping of viruses is typically influenced by variations in antigenic epitopes within their sequences, especially the spike protein [15]. Therefore, we then investigated the relationship between IBV S protein and its serotypes. 110 viruses were initially serotyped, identifying 20 distinct IBV serotypes. Among these, serotypes of QX, Mass, 4/91, TW, and Arkansas were the most prevalent, collectively accounting for 79 of the 110 sequences (Figure 6A). After predicting the T cell and B cell epitopes on the S protein of these 79 sequences, we converted the top 20 scoring epitopes into a position matrix. Using ML, we explored the correlation between the positions of these epitopes and the corresponding serotypes (Figure 6B). The classification of XGBoost revealed distinct epitope patterns across five serotypes (Figure 6C). To independently validate our serotype prediction models, we experimentally determined the serotypes of an additional 11 sequences, which were used to construct an independent validation set. The model demonstrated a high accuracy of 0.91 in predicting serotypes on this validation set (Figure 6D). The model correctly classified 10 sequences belonging to serotypes QX, Mass, 4/91, and TW, and one sequence that did not belong to any of these serotypes. There was a single instance of ambiguity where a TW sequence was predicted as either QX or TW. To conclude, the high predictive accuracy of the model suggests that IBV serotypes can be inferred from epitope positions within the spike protein using our ML framework.

**FIGURE 6:**
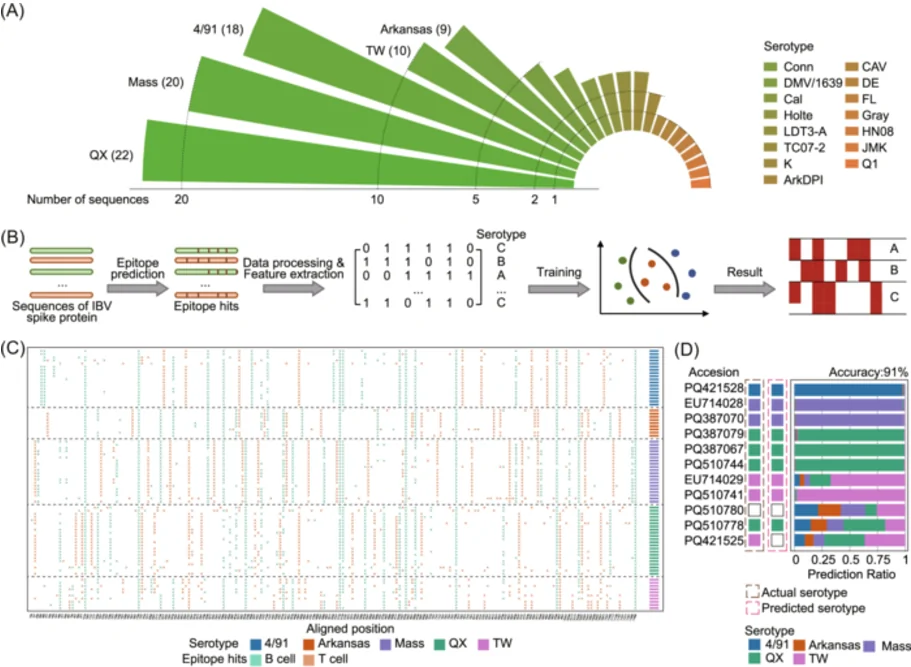
Machine learning models elucidating the relationship between viral sequences and their corresponding serotypes. (A) Distribution of serotypes among 110 sequences analyzed in this study. (B) The schematic representation of ML methodology employed. T and B cell epitope sites on each S protein were initially predicted to extract sequence features. The top 20 scoring sites for each epitope type were selected and converted into a binary matrix (0/1) based on the presence or absence of the epitope at each site. Subsequently, 79 sequences with serotype (4/91, Arkansas, Mass, QX, and TW) information were divided into training and testing sets for model development and evaluation. (C) T and B cell epitope sites on the S protein of 79 sequences representing five distinct serotypes. (D) Stacked bar plot depicts the predicted probabilities for serotype prediction for 11 strains in the validation set. All ML analyses and results presented in this figure were obtained using the XGBoost algorithm.

## 3 DISCUSSION

This study provides a broad epidemiological comparison of six major avian viruses across China from 2003 to 2023, and highlights IBV as the most persistent threat to the poultry industry, which is consistent with previous reports [16, 17]. During this period, we sequenced 136 novel IBV isolates from 14 provinces in China, representing over 25% of full‐length IBV genomes available in NCBI and creating one of the largest field‐derived genomic datasets to date [18, 19]. Combined with corresponding clinical phenotype data, this resource offers a valuable platform for future studies on evolution, diagnostics, and biocontrol, not only of IBV but also of other coronaviruses in general. Furthermore, this study serves as a useful model for understanding the dynamics of coronavirus in broader contexts, including zoonotic spillover and livestock biosecurity [20].

To examine the evolutionary patterns of IBV, we analyzed a total of 624 complete genomes. These include newly discovered field strains along with publicly available strains, and thus, cover a broad range of time points and geographic regions. Phylogenetic reconstruction showed weak regional clustering, especially near the tree's basal nodes, which was dominated by Chinese and North American strains. While this pattern may reflect sampling density, it also suggests that both regions, due to their well‐developed poultry industries, were early contributors to IBV detection [21, 22]. Genome‐wide similarity across regions further supports the notion that IBV exhibits spatial complexity with minimal geographic structuring, a pattern observed in several other livestock viruses [23, 24, 25]. Evolutionary rate analysis estimated a global substitution rate (~4.23 × 10^−4^ substitutions/site/year) comparable to other coronaviruses [25]. When analysis was performed based on the S1 gene, the Chinese IBV strains showed substitution rates similar to those reported in previous studies [26, 27]. However, marked regional variations were evident: viruses from Africa and parts of Asia evolved nearly an order of magnitude faster than those from Europe, the Americas, or Australia, likely due to variations in poultry density, vaccine practices, and biosecurity standards [28]. In contrast, although the Americas and Europe showed lower substitution rates, the broad divergence and confidence intervals suggested complex, multi‐lineage histories. In the United States, long‐term circulation of multiple strains (e.g., Mass, Arkansas, and Connecticut) has contributed to sustained viral diversity [21, 29, 30]. Similarly, in Europe, regional lineages have emerged and become dominant [31, 32, 33]. Hence, these patterns suggest an interplay between ecological and human factors in shaping the global evolution of IBV.

Given the complexity of whole‐genome phylogeography and to elucidate fine‐grained molecular evolutionary patterns, we focused further analyses on the S protein, a highly N‐glycosylated surface protein crucial for viral entry and immune evasion [9, 11, 13, 14]. N‐glycosylation enhances S protein stability and conceals immunogenic epitopes. We thus investigated the evolutionary dynamics of N‐glycosylation motifs to uncover their roles in viral adaptation. Analysis of 10 glycosylation sites in the NTD identified three dominant haplotypes (H9, H12, and H18), characterized by residues at positions 51 and 77, collectively accounting for 86% of the isolates. These haplotypes showed temporal stratification with H12 and H9 appearing earlier, reflecting their basal position in the phylogeny. To test for single origin versus independent origins of the dominant haplotypes, their states were mapped onto the whole‐genome phylogeny. A polyphyletic distribution would support the hypothesis that these two specific amino acid sites are under natural selection. Besides, structural modeling and docking analyses suggested that variation at these two sites alters receptor‐binding potential. H12 (e.g., M41) forms a canonical binding pocket (Asn51/Asn77), whereas H18 shows enhanced affinity via alternative residues (Lys51, Asn75, Ser77). In contrast, H9 lacks a functional binding interface. These structural differences correspond with clinical trends. H9 strains showed lower positivity rates but higher mortality, suggesting a potential trade‐off between transmission efficiency and virulence. However, as these field data are derived from a complex epidemiological landscape subject to numerous confounding variables, experimental validation is required to confirm this association. Despite these limitations, these findings support the idea that glycosylation motif positioning modulates receptor engagement [14, 34]. Notably, previous mutagenesis studies introducing Asn‐to‐Ala substitutions at analogous glycosylation motifs have experimentally abolished IBV's ability to bind the sialoside receptors, providing molecular validation for our structural predictions [35]. In addition, such associations align with findings from SARS‐CoV and influenza virus, where glycosylation changes influence receptor usage and pathogenicity [36, 37]. By contrast, glycosylation sites near the furin cleavage region (positions 542 and 686) were highly conserved and showed no clear link to clinical severity. Nonetheless, previous studies suggest that Asn‐to‐Ser substitutions at analogous sites in SARS‐CoV‐2 can introduce O‐glycosylation motifs, forming glycan shields that impair furin cleavage [38]. Similarly, IBV variants (e.g., N542S/T) have been experimentally linked to altered viral entry [39]. Although AlphaFold‐based protein structural prediction does not explicitly model glycans, our modeling also suggests potential impacts on epitope exposure or protease accessibility, which warrants further investigation [40]. For example, studies should leverage established IBV reverse genetics systems to introduce these key mutations, providing the causal evidence necessary to confirm that these glycosylation motifs are, in fact, key virulence determinants.

Strikingly, the evolutionary patterns of the NTD glycosylation haplotypes appear to underpin the large‐scale spatiotemporal dynamics. For instance, the emergence and increasing prevalence of the H18 haplotype (Figure 3C), which our structural analysis predicts to have enhanced the sialoside receptor binding affinity (Figure 4D), coincides with the period of rapid global transmission within the core triangle observed from 2011 to 2017 (Figure 5C). It is tempting to speculate that this structural innovation conferred a fitness advantage, such as more efficient transmission, that facilitated this global spread. In contrast, the H9 haplotype, associated with higher mortality but lower positivity, may represent a different adaptive strategy. This direct link between a defined molecular feature (glycosylation motif) and a macro‐evolutionary outcome (global dissemination) illustrates the power of an integrated genomic‐structural‐phenotypic framework. This approach moves beyond simple tracking to provide mechanistic hypotheses for why certain lineages succeed and spread. Besides, our data confirm that IBV transmission has been centered for a long time on a tri‐regional axis comprising North America, Europe, and East Asia (especially China). This pattern aligns with the initial phase of poultry industry globalization and a lack of standardized vaccination protocols across continents [41]. The subsequent period of relative stability (2008–2011) may correspond to the broad deployment of Mass‐type vaccines, which could have temporarily suppressed viral expansion [22]. A marked shift occurred between 2011 and 2017, characterized by wider dissemination to South America and Africa. This expansion coincided with the growth of poultry markets, increasing global connectivity, and increased international trade in live birds—key drivers of pathogen spread [23, 28]. After 2017, the refocus of transmission within the core triad likely reflects updates to regional vaccine strategies and enhanced biosecurity in previously affected regions. Within China, the evolving spread of IBV over decades has similarly been influenced by shifts in farming practices, vaccine application, and industry infrastructure [42].

Beyond tracing the spatiotemporal dissemination of IBV, a critical challenge in the field remains the rapid and accurate determination of serotypes for timely vaccine matching and clinical management [43]. The spike protein, as the primary target of neutralizing antibodies and a key determinant of cell‐mediated immunity [44, 45], logically serves as the basis for serotype prediction [46, 47, 48]. To address this, we developed a novel machine learning model that leverages T‐cell and B‐cell epitope features within the S protein to predict clinical serotypes, achieving a high validation accuracy of 91%. This demonstrates a strong association between S protein sequence features and serotype, offering a valuable complement to labor‐intensive serological assays. While the model's current scope is trained on a defined subset of prevalent serotypes and its generalizability to all circulating strains requires continuous validation, it establishes a foundational and accessible framework for sequence‐based serotyping. Future efforts to expand the training dataset will undoubtedly enhance its resolution and pave the way for even more powerful, deep learning‐based approaches [49, 50], ultimately enabling more proactive surveillance and control strategies.

In conclusion, this study presents one of the most comprehensive genomic and phenotypic investigations of IBV to date, integrating large‐scale field surveillance, evolutionary genomics, structural modeling, serotypic prediction, and spatiotemporal transmission dynamics. As coronaviruses continue to diversify, sustained genomic surveillance combined with the integrated analytical framework will be essential for anticipating emerging threats and guiding future control strategies in both veterinary and broader coronavirus research contexts.

## 4 CONCLUSION

By integrating genomics, structural biology, and machine learning, this work constructs a predictive framework for coronavirus evolution. We demonstrate that glycosylation haplotypes in the spike protein serve as evolutionary and epidemiological markers, may contribute to receptor binding, clinical severity, and transmission. Simultaneously, our computational model enables highly reliable serotype prediction directly from viral sequence data. Together, these findings provide a conceptual framework for anticipating viral adaptive trajectories, with direct implications for vaccine design and preemptive pandemic preparedness.

## 5 METHODS

### Sample collection, virus isolation, and sequencing

From 2003 to 2023, a nationwide surveillance effort was conducted across 29 provinces in mainland China (excluding Tibet, Hainan, Hong Kong, Macau, and Taiwan), involving millions of diagnostic screenings for six major avian pathogens: infectious bronchitis virus (IBV), avian influenza virus (AIV), infectious bursal disease virus (IBDV), avian reovirus (ARV), infectious laryngotracheitis virus (ILTV), and Newcastle disease virus (NDV). Clinical samples, including throat and cloacal swabs, kidneys, spleens, bursae of Fabricius, and oviducts, were collected from symptomatic poultry and detected by reverse transcription‐polymerase chain reaction (RT‐PCR) or quantitative real‐time polymerase chain reaction through virus‐specific primers (Table S7). This multifaceted approach enabled annual estimation of both positivity and mortality rates for each pathogen (Table S1). For IBV, 67 strains were linked to clinical outcomes, including field‐level positivity and mortality rates (Table S2).

Specific pathogen‐free (SPF) chicken embryos were utilized to propagate IBV. The allantoic fluid was collected from infected embryos at 48 to 72 h post‐infection. The virus was subsequently isolated through ultracentrifugation using a pH 7.2 HCl‐PBS buffer containing 30% (w/v) sucrose. RNAs from plaque‐purified viruses were extracted using the QIAamp Viral RNA Kits (Qiagen, 52904). The cDNA was synthesized using random primers and the RevertAid First Strand cDNA Synthesis Kit (Thermo Scientific, K1622). Each sample was further confirmed by RT‐PCR, followed by viral isolation/purification, and was subsequently sequenced using the Illumina HiSeq. 2500 platform with paired‐end reads of 150 bp (PE150). The short reads of each sample were de novo assembled following filtering out the host sequence. All 136 sequences have been deposited in the GenBank database (Table S2).

### Data acquisition, curation, and quality control

All publicly available complete genome sequences of IBV, as of September 4, 2023, were retrieved from NCBI GenBank (https://www.ncbi.nlm.nih.gov/). After excluding vaccine strains, experimentally attenuated strains, sequences with unclear annotations for year and location, and those with sequence length >30,000 bp, a total of 624 sequences were retained, including 136 newly originated from this study (Table S2).

Using MUSCLE v3.8.31 [51], all 624 sequences were aligned. Subsequently, the Python package SeqKit [52] was employed to split the completely aligned sequences into individual protein nucleotide sequences that include non‐structural proteins (pp1ab), accessory proteins (gene 3a/b, gene 5a/b), and structural proteins [(spike (S), envelope (E), membrane (M), and nucleocapsid (N)].

### Phylogeny, similarity and evolutionary rate estimation, and recombination analysis

A maximum likelihood phylogenetic tree was inferred from 624 complete IBV genome sequences using FastTree v2.1.10 [53], applying the general time reversible substitution model with gamma‐distributed rate heterogeneity. Statistical support was assessed through 1,000 bootstrap replicates. The resulting phylogeny was visualized and annotated using the Interactive Tree of Life (iTOL; https://itol.embl.de). For regional comparisons, all strains were stratified into seven geographic groups: China, Europe, North America, South America, other Asian countries, Africa, and Australia. Pairwise sequence similarities were subsequently calculated for both whole genomes and individual coding regions, including polyproteins (ORF1a and ORF1b) and structural proteins, and summarized by geographic region. To explore evolutionary dynamics, time‐scaled root‐to‐tip divergence was estimated using TreeTime [12]. Recombination events were identified within each regional subset using the Recombination Detection Program v5 (RDP5) [54]. Only recombination events supported by at least three of the seven algorithms implemented in RDP5 (with *p* < 0.05) were retained. Recombinant sequences were removed iteratively until no additional events were detected.

### Haplotype dynamics analysis

To investigate the evolutionary variation of N‐linked glycosylation in the S protein, we performed haplotype analysis based on predicted N‐glycosylation sites. Full‐length S gene sequences were translated in silico and aligned using MUSCLE v3.8.31. One strain, ahysx‐1 (GenBank accession Number MK142676), exhibited substantial divergence in its S protein sequence compared to other strains and was therefore excluded from subsequent analyses. 10 representative IBV strains with NCBI accession numbers of AY338732, DQ834384, FJ904717, JQ977698, KP118885, KT852992, KY799582, MK581202, MN128088, and KX302860 were selected for glycosylation site prediction using the NetNGlyc 1.0 server (https://services.healthtech.dtu.dk/services/NetNGlyc-1.0/) [55]. Prediction criteria followed the default thresholds defined by the server. Prediction only on Asparagines (Asn) that occur within the Asn‐X‐Ser/Thr triplet were involved in the following analysis. The union of predicted N‐glycosylation sites across these 10 strains yielded a consensus set of 41 Asparagine sites, corresponding to conserved Asn‐X‐Ser/Thr motifs. Among them, 10 sites were localized to the NTD and 14 to the S2 subunit. The remaining 17 are situated in other regions of the spike protein outside the NTD and S2 subunit and were excluded from subsequent haplotype analyses. In this study, glycosylation haplotype analysis was performed separately for the 10 Asn sites in the NTD (NTD haplotypes) and the 14 Asn sites in the S2 subunit (S2 haplotypes). Haplotype inference based on the presence or absence of these N‐linked glycosylation sites across the 623 remaining S protein sequences was performed using DnaSP v6.0 [56]. A Templeton, Crandall, and Sing network was constructed using POPART [57, 58], and the resulting haplotype network was refined and visualized using Cytoscape v3.8.2 [59].

### Protein structure analysis and receptor docking analysis

Structural models of S proteins from five representative IBV strains, including M41 (GenBank accession Number: DQ834384), ck/CH/LSD08‐7 (KX302860), ck/CH/LSD/110857 (KP118885), gammaCoV/Ck/Poland/80/1989 (MK581202), and ck/CH/LSD/111219 (KJ435283), were generated via protein structure prediction using AlphaFold3 (https://alphafoldserver.com/). Resulting protein structures were visualized and analyzed using PyMOL (v2.0, Schrödinger, LLC; https://pymol.org/2/). The 3D structural file of the receptor Neu5Acα2‐3Galβ1‐3GlcNAc was retrieved from the PubChem database (https://pubchem.ncbi.nlm.nih.gov/). Before molecular docking, hydrogen atoms were added, and the structural files for the Neu5Acα2‐3Galβ1‐3GlcNAc and three selected S protein models (M41, ck/CH/LSD08‐7, and ck/CH/LSD/110857) were preprocessed in PyMOL. Docking analyses were performed using AutoDockTools v4.2.6 [60]. Grid box parameters were defined to encompass the entire NTD of the S protein, and docking was executed with default parameters, except for the number of genetic algorithm (GA) runs, which was set to 50. Docking outputs were visualized in PyMOL for structural interpretation of receptor‐binding interfaces.

### Time‐calibrated phylogeographic reconstruction of IBV

Bayesian evolutionary and phylogeographic analyses were performed using the BEAST2 [61]. Parameter estimates from BEAST2 were analyzed using Tracer v1.7 [62], with chain lengths set to 800 million to ensure convergence. All estimated parameters from BEAST2 were based on an effective sample size (ESS) greater than 200. For the analysis and visualization of pathogen phylogenetic reconstructions, we employed spread.gl (Spatial Phylogenetic Reconstruction of Evolutionary Dynamics built on kepler.gl; https://beast.community/spreadgl). Thus, the detailed time‐calibrated phylogeographic maps, revealing the global and China‐specific transmission patterns of IBV, were reconstructed.

### Machine learning classifier model for serotype prediction

The virus neutralization test is by far the most accurate method available for differentiating between IBV serotypes as well as for confirming the identity of new ones. According to Cook et al., we performed the tests to validate the serotypes of IBV strains [63]. A total of 110 sequences with serotype information were acquired. The five most prevalent serotypes were QX (22 sequences), Mass (20), 4/91 (18), TW (10), and Arkansas (9), collectively amounting to 79 sequences. This subset of 79 sequences was selected for constructing the machine learning‐based serotype prediction classifier (Figure 6A, Table S2). We then extracted the protein sequences of their S proteins. Utilizing the CTLPred tool [64], we employed an Artificial Neural Network (ANN) approach to predict T‐cell epitopes, setting the number of peptide segments with a window length of 9 and the ANN cutoff score to the default value of 0.51. Concurrently, we utilized the ABCpred tool [65] to predict B‐cell epitopes, with the threshold and window length also set to 0.51 and 16, respectively, in accordance with default settings. For both T‐cell and B‐cell epitope predictions, we ranked the epitopes for each strain based on their scores in descending order, selecting those with scores that placed them within the top 20 or above (in cases where multiple epitopes shared the same score). We then compiled the positions of all peptide segments identified as potential epitopes across different strain sequences. A binary matrix was constructed with strain names on the *y*‐axis and peptide position intervals arranged in ascending order on the *x*‐axis. In this matrix, a value of 1 was assigned to indicate the presence of an epitope at a specific position interval in a given strain's S protein sequence, whereas a value of 0 was assigned otherwise. Using this data matrix, we employed the XGBoost algorithms to construct a predictive classification model for serotype identification, randomly partitioning the results of the 79 sequences (serotype of 4/91, Arkansas, Mass, QX, and TW) into training and testing datasets in an 8:2 ratio. The source code and implementation details of the model are publicly available at: https://github.com/Azzurro117/Zhang2025IMO. Finally, we established an independent validation set by determining the serotypes of 11 additional sequences via virus neutralization test (Table S2); these sequences were not part of the initial 110 sequences. Their NCBI accession numbers and assigned serotypes are: EU714028 (Mass), EU714029 (TW), PQ387067 (QX), PQ387070 (Mass), PQ387079 (QX), PQ421525 (TW), PQ421528 (4/91), PQ510741 (TW), PQ510744 (QX), PQ510778 (QX), and PQ510780 (unknown).

### Statistical analysis and visualization

Unless otherwise stated, all statistical analyses and visualizations were performed using R version 4.4.1. The graphical representations and formatting were performed utilizing R version 4.4.1 and GraphPad Prism 8.0 in conjunction with Adobe Illustrator 2024.

## AUTHOR CONTRIBUTIONS

**Hao Zhang**: Conceptualization; software; data curation; resources; writing—review and editing; writing—original draft; investigation; methodology; visualization. **Zongxi Han**: Data curation; resources; investigation; methodology; funding acquisition. **Chuangchao Zou**: Software; methodology; writing—original draft; writing—review and editing; visualization; formal analysis. **Yihui Huang**: Software; methodology; writing—original draft; visualization; formal analysis. **Shiping Sun**: Data curation; resources; investigation; methodology; formal analysis. **Ouyang Peng**: Investigation; data curation. **Usama Ashraf**: writing—original draft. **Qiuping Xu**: writing—original draft. **Yao Ge**: Investigation; methodology. **Qixiang Kang**: Investigation; methodology. **Xiuli Ma**: Investigation; methodology. **Xinheng Zhang**: Investigation; methodology. **Ye Zhao**: Investigation; methodology. **Xin Yang**: Conceptualization; resources. **Hongning Wang**: Resources. **Guozhong Zhang**: Conceptualization; resources. **Qingmei Xie**: Conceptualization; resources. **Mang Shi:** Writing—review and editing. **Yongsen Ruan**: Software, data curation; writing—review and editing; formal analysis. **Min Liao**: Conceptualization; resources; funding acquisition. **Shengwang Liu**: Conceptualization; resources. **Yongchang Cao**: Funding acquisition; investigation; conceptualization; methodology; resources; writing—review and editing; validation; supervision; project administration. All authors have read the final manuscript and approved it for publication.

## CONFLICT OF INTEREST STATEMENT

The authors declare no conflicts of interest.

## ETHICS STATEMENT

The animal studies were approved by the Animal Ethics Committee of Harbin Veterinary Research Institute (HVRI) of the Chinese Academy of Agricultural Sciences (CAAS) (240710‐01‐GR) and implemented in accordance with regulation and guidelines of this committee.

## Supporting information


**Figure S1.** Overview of 136 newly sequenced whole‐genome IBV strains from China isolated in this study.
**Figure S2.** Pairwise nucleotide similarity matrix of six IBV genes (ORF1a, ORF1b, spike, envelope, membrane, nucleocapsid) across seven geographic regions globally.
**Figure S3.** Root‐to‐tip genetic distances of seven geographic regions plotted against sampling dates, along with the estimate of substitution rates.
**Figure S4.** Breakpoint distributions of recombination events in IBV strains of seven geographical regions.
**Figure S5.** A median‐joining haplotype network was constructed from 623 IBV S protein sequences based on 10 N‐linked glycosylation sites (related to Fig. 3B).
**Figure S6.** Evolutionary diversification of N‐linked glycosylation haplotypes in the S2 subunit of IBV S protein.
**Figure S7.** Alphafold3‐based predicted structural models of the S protein for strains.
**Figure S8.** Spatiotemporal dissemination of IBV strains in China.

**Table S1.** Investigated information of six avian viruses in China during 2003 to 2023.
**Table S2.** The information of 624 IBV strains used in our study.
**Table S3.** Estimated evolutionary rates of different sets of IBV strains using Treetime.
**Table S4.** Information of recombination events using RDP5.
**Table S5.** Haplotype information of N‐glycosylation sites in NTD and S2 of spike protein.
**Table S6.** Prediction of N‐glycosylation sites of spike protein of 10 IBV strains using NetNGlyc.
**Table S7.** Primers used in RT‐PCR or qPCR for virus detection.

## Data Availability

All 136 sequenced strains in the study are available in the National Center for Biotechnology Information (NCBI) with GenBank accession numbers PQ387060 – PQ387079, PQ421521 – PQ421569, and PQ510737 – PQ510803 (Table S2). The data and scripts used are saved in GitHub https://github.com/Azzurro117/Zhang2025IMO. Supplementary materials (figures, tables, graphical abstract, slides, videos, Chinese translated version, and update materials) may be found in the online DOI or iMeta Science http://www.imeta.science/imetaomics/.
