# Supplementary material for: The evolutionary landscape and serotypic dynamics of avian infectious bronchitis virus from spike protein
Source: IMetaOmics. 2026 Jan 19;3(1):e70077. doi: 10.1002/imo2.70077 (PMC13674424; doi:10.1002/imo2.70077)
Supplement: Supplementary file 1 [file IMO2-3-e70077-s002.docx]

Supporting information to

**The Evolutionary Landscape and Serotypic Dynamics of Avian Infectious Bronchitis Virus from Spike Protein**

Running title: Evolutionary Landscape and Serotypic Dynamics of IBV from Spike Protein

Hao Zhang^1#^, Zongxi Han^2#^, Chuangchao Zou^1#^, Yihui Huang^1#^, Shiping Sun^3#^, Ouyang Peng^1^, Usama Ashraf^4^, Qiuping Xu^5^, Yao Ge^3^, Qixiang Kang^1^, Xiuli Ma^6^, Xinheng Zhang^7^, Ye Zhao^8^, Hongning Wang^9^, Xin Yang^9*^, Guozhong Zhang^8^*, Qingmei Xie^7^*, Mang Shi^10^*, Yongsen Ruan^1^*, Min Liao^3^*, Shengwang Liu^2^*, Yongchang Cao^1^*

^1^State Key Laboratory of Biocontrol, School of Life Sciences, Sun Yat-sen University, Guangzhou 510275, China

^2^State Key Laboratory of Animal Disease Control and Prevention, Harbin Veterinary Research Institute, Chinese Academy of Agricultural Sciences, Harbin 150001, China.

^3^Key Laboratory of Animal Virology of Ministry of Agriculture, Zhejiang University, Hangzhou 310058, China.

^4^Programme in Emerging Infectious Diseases, Duke-NUS Medical School, Singapore 169857, Singapore.

^5^Guangdong Provincial Key Laboratory of Malignant Tumor Epigenetics and Gene Regulation, Sun Yat-sen Memorial Hospital, Sun Yat-sen University, Guangzhou 510120, China.

^6^Shandong Academy of Agricultural Sciences, Jinan 250100, China.

^7^College of Animal Science, South China Agricultural University, Guangzhou 510642, China.

^8^College of Veterinary Medicine, China Agricultural University, Beijing 100193, China.

^9^College of Life Sciences, Sichuan University, Chengdu 610064, China.

^10^Key Laboratory of Intelligent Tracking and Forecasting for Infectious Diseases, State Key Laboratory for Biocontrol, School of Medicine, Shenzhen Campus of Sun Yat-sen University, Sun Yat-sen University, Shenzhen 518107, China.

^#^These authors contributed equally.

*Correspondence: [caoych@mail.sysu.edu.cn](mailto:caoych@mail.sysu.edu.cn) (Yongchang Cao)

**Supplementary figure and figure legend**

**Figure S1. Overview of 136 newly sequenced whole-genome IBV strains from China isolated in this study.**

(A) Temporal distribution of sequenced strains. The grey bars indicate the yearly number of sequenced strains. whereas the red line indicates accumulative number of sequenced strains. (B) Geographic provenance of sequenced strains. Province labels indicate sample counts (number shown in parentheses).

**Figure S2. Pairwise nucleotide similarity matrix of six IBV genes (ORF1a, ORF1b, spike, envelope, membrane, nucleocapsid) across seven geographic regions globally.** The corresponding mean, minimum, and maximum values were calculated from all pairwise sequence comparisons between the two respective geographic regions.

**Figure S3. Root-to-tip genetic distances of seven geographic regions plotted against sampling dates, along with the estimate of substitution rates.** The strains (each dot represents a strain) in the outer plots were included in the linear fitting the evolutionary rate estimation according to TreeTime[53]. The inner plots show the posterior probability densities of the mean estimated evolutionary rate and the *x*-axis represents the evolutionary rate (×10^−4^ substitutions per site per year). Colors correspond to the seven geographic regions, following the same color scheme as in Figure 2A.

**Figure S4. Breakpoint distributions of recombination events in IBV strains of seven geographical regions.** Each square represents one recombination breakpoint. Colors correspond to the seven geographic regions, following the same color scheme as in Figure 2A.

**Figure S5. A median-joining haplotype network was constructed from 623 IBV S protein sequences based on 10 N-linked glycosylation sites** (related to Fig. 3B). Colored labels next to each haplotype denote the earliest recorded sampling year in each of the seven geographic regions and text color corresponds to regional origin.

**Figure S6. Evolutionary diversification of N-linked glycosylation haplotypes in the S2 subunit of IBV S protein.** (A) Amino acid distribution across 14 predicted N-linked glycosylation sites in the S2 subunit of IBV S protein. All annotated positions are relative to the M41 reference strain (GenBank accession Number DQ834384). (B) Median-joining haplotype network constructed from 623 IBV S sequences based on the 14 N-linked glycosylation sites. Each circle represents a unique haplotype; circle size indicates the number of sequences (number shown in parentheses if > 1), and colors denote geographic origins. Edge thickness corresponds to the number of amino acid substitutions between haplotypes. (C) Comparison of clinical positivity rates and mortality outcomes among three major haplotype groups differentiated by their amino acid combinations at positions 542 and 686. Statistical comparisons were conducted using the Kolmogorov–Smirnov test; *p*-values < 0.05 are annotated. Exact values are listed in Table S2.

**Figure S7. Alphafold3-based predicted structural models of the S protein for strains.** (A) gammaCoV/Ck/Poland/80/1989 (GenBank accession Number. MK581202), (B) ck/CH/LSD/110857 (KP118885), and (C) ck/CH/LSD/111219 (KJ435283). NTD, N-terminal domain. CTD, C-terminal domain. FP: fusion peptide. HR1 and HR2: heptad repeats 1 and 2. CH: central helix.

**Figure S8. Spatiotemporal dissemination of IBV strains in China.** The Bayesian stochastic search variable selection approach was used to elucidate the spatiotemporal dynamics of IBV dissemination. The intensity of inferred transmission events, supported by a Bayes Factor (BF) exceeding three, is visualized. Color-coding differentiates transmission events occurring across four distinct temporal periods from 1980 to 1997 (A), 1997 to 2007 (B), 2007 to 2017 (C), and 2017 to 2023 (D). The variation in circle size is set for visual distinction and carries no quantitative meaning regarding the geographic data.
